# Abscission zones: cellular interfaces for the programmed separation of organs

**DOI:** 10.1093/aob/mcaf034

**Published:** 2025-03-06

**Authors:** Véronique Pautot, Jennifer Crick, Shelley R Hepworth

**Affiliations:** Université Paris-Saclay, INRAE, AgroParisTech, Institute Jean-Pierre Bourgin for Plant Sciences (IJPB), 78000, Versailles, France; Department of Biology, Carleton University, Ottawa, Ontario K1S 5B6, Canada; Department of Biology, Carleton University, Ottawa, Ontario K1S 5B6, Canada

**Keywords:** Abscission zone, *Arabidopsis*, floral organ abscission, tomato, pedicel abscission, auxin, ethylene

## Abstract

**Background:**

Abscission zones are specialized sites where plants shed organs, such as leaves, petals or fruits, in response to developmental or environmental signals. These zones form at predictable locations and, once activated, undergo structural and physiological changes that detach the organ and seal the exposed area. During crop domestication, plants that retained ripe fruit or seeds were selected, and abscission traits still influence crop yield and quality today.

**Scope:**

This article reviews the stages of development of abscission zones: initiation, competence, separation and sealing. We combine insights from classic structural and physiological studies with modern genetic and molecular research, focusing on two plant species: *Arabidopsis thaliana* as a model for floral organ abscission and *Solanum lycopersicum* as a model for fleshy fruit development.

**Conclusions:**

These studies show that abscission is a conserved but flexible developmental process. We conclude by exploring how these findings are being applied to improve abscission traits in modern agriculture.

## INTRODUCTION

Plants shed organs through a process called abscission. Abscission takes place at specialized sites called abscission zones (AZs) that control the precise separation of organs from aerial parts of the plant. This ability allows plants to disperse offspring or adjust their structure during development or in response to stresses (Addicott, 1982; Kim *et al.*, 2019; Patharkar and Walker, 2019; Ma *et al.*, 2021).

Abscission is an ancient process. In mosses, it enables vegetative reproduction by gemmae detachment or spore dispersal by release of a capsule lid, the operculum (Addicott, 1982; Duckett and Ligrone, 1992). In the peat moss *Sphagnum*, this release is explosive, driven by tension from the drying and shrinking capsule wall (Ingold, 1965; Addicott, 1982). As land plants evolved, cell separation became targeted to other precise locations, enabling the selective removal of organs at particular life stages or in response to environmental signals (Addicott, 1991). A familiar example is the seasonal shedding of autumn leaves. In Japan, the autumn season draws millions of visitors each year to witness the spectacular changing of leaf colour, known locally as koyo and momiji. Some species, such as the invasive African wood sorrel (*Oxalis pes-caprae*), have lines of weakness that allow leaves to detach easily under stress as a defence mechanism, resembling autotomy, a form of self-amputation, in animals (Shtein *et al.*, 2019). Abscission also aids in seed and fruit dispersal, allowing plants to reproduce and colonize new territories (Patterson *et al.*, 2016). Dehiscence, a related process, involves the splitting of mature plant structures along seams called dehiscence zones (DZs). For example, seeds are released by this mechanism in crucifers and legumes, whose dried pods split open under tension (Dong and Wang, 2015; Ballester and Ferrándiz, 2017). Abscission and dehiscence are controlled by distinct sets of regulatory genes but share some common features (Patharkar and Walker, 2019). Both processes lead to the formation of a separation layer and, often, a lignified layer (Lee *et al.*, 2018) and involve common cell wall remodelling proteins and enzymes for cell separation (Ballester and Ferrándiz, 2017).

Abscission and dehiscence can cause crop losses when seeds or fruits are shed before harvest but can also be useful. In tree crops, growth regulators thin young fruits to control fruit load (Bangerth, 2000) and aid in mechanical harvesting of hard-to-reach fruit or nuts (Richardson and Dawson, 1993; Burns, 2002). Preharvest leaf abscission is induced to boost yields in cotton and sugarcane (Li *et al.*, 2023). The timing of abscission has long been a focus of breeding in cereal crops, such as rice, maize and wheat. For example, the domestication of Asian rice ~9000 years ago involved the selection of a low seed shattering *sh4* variant in *japonica*, later introduced into *indica* varieties (Sang and Ge, 2013). Reduction of shattering in rice is important, not only to increase grain yield, but also to adapt rice varieties to mechanical harvesting. Although seed and fruit retention has been strongly selected, these traits remain a breeding target owing to limited natural variation (Li and Olsen, 2016; Ogutcen *et al.*, 2018).

**Fig. 1.**
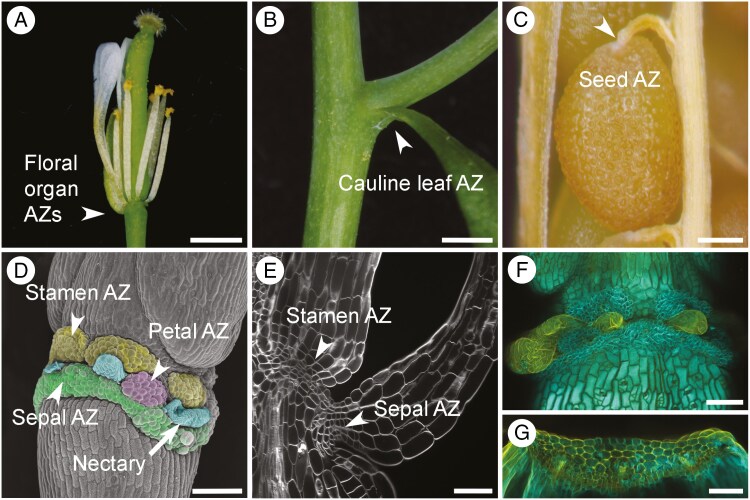
Placement of abscission zones (AZs) in Arabidopsis plants. (A) Flower containing floral organ AZs (arrowhead). Scale bar: 1 mm. (B) Cauline leaf AZ (arrowhead). Scale bar: 4 mm. (C) Seed AZ (arrowhead). Scale bar, 150 μm. (D) Scanning electron micrograph showing the placement of sepal, petal and stamen AZs (arrowheads) and nectary on the fruit receptacle (arrow). Scale bar: 100 μm. (E) Structure of floral organ AZs: maximum projection confocal microscopy images showing sepal and stamen AZ cells (arrowheads). Scale bar: 50 μm. (F) Maximum projection confocal microscopy images showing the wild-type receptacle following floral organ abscission. Scale bar: 100 μm. (G) Maximum projection confocal microscopy images showing the lignin brace structure in sepals. Scale bar: 50 μm. Lignin was visualized by basic fuchsin (orange) staining in combination with Fluorescent Brightener (Calcofluor White M2R) to visualize cell walls (cyan).

**Fig. 2.**
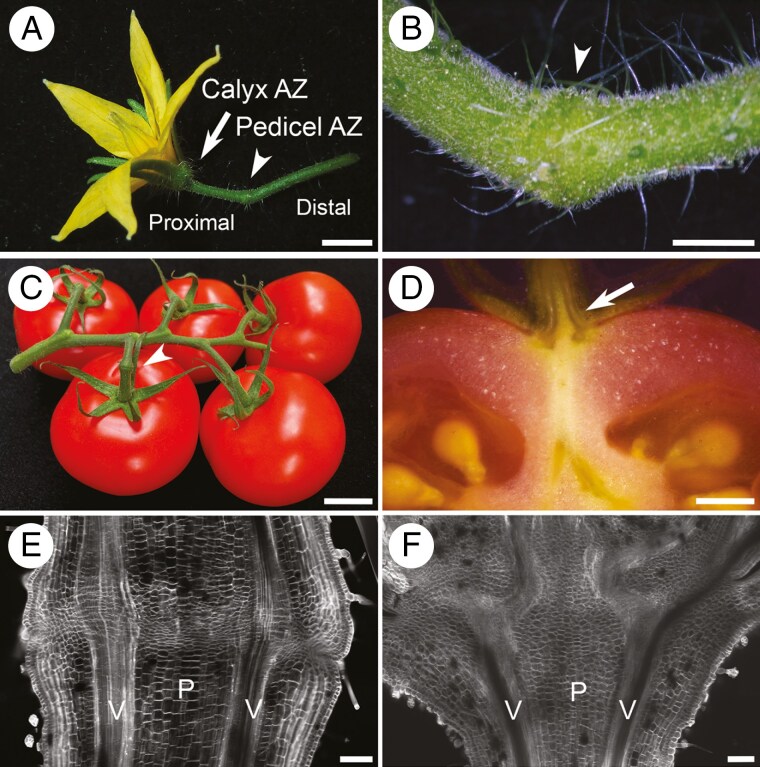
Placement of abscission zones (AZs) in tomato plants. (A) Pedicel AZ at the flower stage (arrowhead) showing proximal and distal domains, calyx AZ (arrow). Scale bar: 1 cm. (B) Pedicel AZ, close-up (arrowhead). Scale bar: 1 mm. (C) Pedicel AZ at the ripe fruit stage (arrowhead). Scale bar: 2 cm. (D) Tomato section showing the calyx AZ (arrow). Scale bar, 2 mm. (E) Confocal microscopy image showing the pedicel AZ. Scale bar: 100 μm. (F) Confocal microscopy image showing the calyx AZ. Scale bar: 100 μm. Abbreviations: P, pith; V, vascular bundle. Samples were stained with Fluorescent Brightener (Calcofluor White M2R) to visualize cell walls (white).

Abscission has been studied for decades, with early work focused on the anatomy and physiology of AZs (Addicott, 1982). Molecular studies have centred on *Arabidopsis thaliana* (Arabidopsis) as a model for floral organ abscission and *Solanum lycopersicum* (tomato) as a model for fleshy fruit development (Patterson, 2001; Roberts *et al.*, 2002; Nakano *et al.*, 2013; Dong and Wang, 2015; Tucker and Kim, 2015; Patharkar and Walker, 2018; Ma *et al.*, 2021; Figs 1 and 2). Rice is often used as a model for grasses (Dong and Wang, 2015; Yu *et al.*, 2020*a*; Wu *et al.*, 2023; Li and Su, 2024). New technologies have expanded abscission studies to more species, including cereals, fruits, vegetables, trees and ornamental plants (Tranbarger and Tadeo, 2020; Shi *et al.*, 2023; Yu and Kellogs, 2024). For example, inhibition of fruit abscission in vine crops reduces spoilage (Périn *et al.*, 2002), and in tree crops, the management of flower and fruitlet abscission controls yield (Merelo *et al.*, 2017; Tranbarger and Tadeo, 2020). In cut-flower crops, such as roses, delaying petal abscission can extend vase life and boost value (Singh *et al.*, 2020). Understanding abscission at a molecular level is a key to modifying this important crop trait. In this review, we mainly focus on Arabidopsis and tomato species but include findings in the tropical fruit *Litchi chinensis* (litchi), for which links to ethylene have been characterized recently.

## STRUCTURE AND PLACEMENT OF ABSCISSION ZONES

Plant species vary widely in the location, number and structure of AZs (Addicott, 1982). The AZs can form in stems, petioles and pedicels but typically form at an organ boundary, a distinct layer of meristematic cells at the base of a lateral organ (Addicott, 1982; Sexton and Roberts, 1982; Osborne and Morgan, 1989). These early-forming or primary AZs differ from secondary or adventitious AZs, which develop later, in mature organs, often near sites of injury (Addicott, 1982; Osborne and Morgan, 1989; Marasek-Ciolakowska *et al.*, 2020).

Typical AZs in dicot species consist of small, tightly packed cells with a dense cytoplasm, arranged in 2–50 cell layers (Sexton and Roberts, 1982). Arabidopsis floral AZs have four to six cell layers (McKim *et al.*, 2008), and the tomato pedicel AZ has six to eight cell layers at flower anthesis (Tabuchi *et al.*, 2001). Cell wall breakdown is generally confined to a one- to five-cell-wide separation layer within the AZ (Sexton and Roberts, 1982). In grasses, the separation layer varies, featuring a disarticulation zone where cells can undergo abscission or mechanical rupture (Yu and Kellogg, 2024).

In Arabidopsis, AZs form at boundaries between the base of floral organs and the receptacle (Bleecker and Patterson, 1997; Stenvik *et al.*, 2006; Fig. 1A, D, E). AZs also form at the base of cauline leaves (Stenvik *et al.*, 2006; Fig. 1B). Most studies focus on floral organs because their abscission is regulated developmentally (Bleecker and Patterson, 1997). Cauline leaf abscission, only recently discovered, is triggered by drought or bacterial infection (Patharkar *et al.*, 2017). AZs also form at the base of the seed and the funiculus, a stalk that connects the seed to the placenta inside the fruit (Pinyopich *et al.*, 2003; Balanzà *et al.*, 2016) and (Fig. 1C).

In tomato, an AZ described as a ‘joint’ forms about midway in the pedicel, a stalk that connects the flower or fruit to the plant (Butler, 1936; Fig. 2A–C, E). The AZ initiates during development of sepal primordia in central tissues of the pedicel and extends gradually to the surface (Tabuchi *et al.*, 2001; Liu *et al.*, 2014). Clonal analysis showed that meristematic cells in the pedicel core influence the fate of surrounding cells to form the AZ (Szymkowiak and Irish, 1999). Detachment points also exist at the boundary between the tomato fruit and the calyx for the release of floral organs and ripe fruit (Butler, 1936; Tabuchi and Arai, 1999; Fig. 2A, D, F). Likewise, AZs at the petiole base enable leaf detachment.

Abscission zones can form in different locations at a boundary or within the tissues. In citrus, as in tomato, pedicel AZs allow flowers on individual stems to separate off the main branch without following a boundary. Fleshy fruit species can have up to three AZs, usually at the fruit base and the pedicel or within the pedicel itself (Tranbarger and Tadeo, 2020). Others are located at the boundary between the pedicel and the peduncle or rachis or bract tissues. These AZs can have distinct activities. For example, a single AZ can function for flower and fruit abscission, as in apple, whereas other crops have multiple AZs that operate sequentially either for flower and fruitlet abscission or for mature fruit abscission. Grasses also show complex AZ patterns (Yu and Kellogg, 2018, 2024; Yu *et al.*, 2020*a*, *b*). Depending on the species, AZs can form in the branch or rachis, in the pedicel or beneath each floret (Yu *et al.*, 2020*b*).This diversity is associated with distinct gene regulatory networks (Yu and Kellogg, 2018, 2024; Yu *et al.*, 2020*a*, *b*).

## FOUR STEPS OF ABSCISSION

**Fig. 3.**
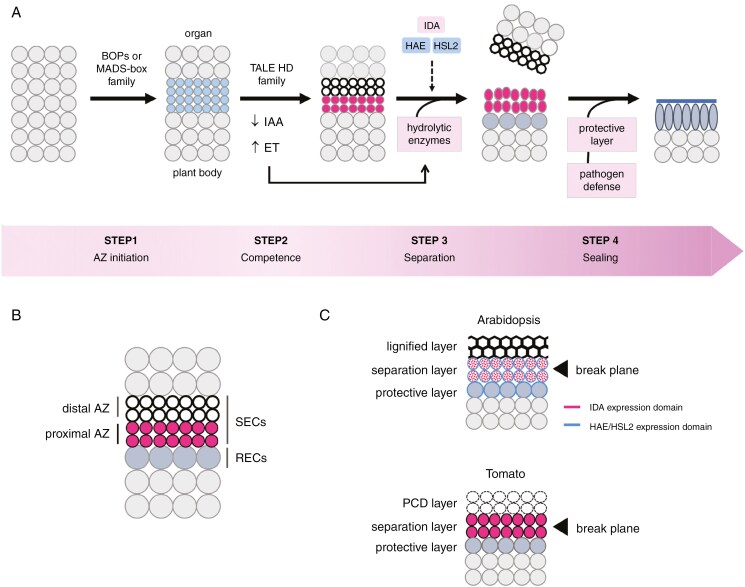
Physiological and genetic model of abscission. (A) Four-step process of abscission. In step 1, abscission zones (AZs) are initiated at specific sites as layers of small dense cells (small blue circles). BOPs are required for initiation at leaf and floral organ boundaries, whereas MADS-box factors are essential for initiation in pedicels and seeds. In step 2, the AZ becomes competent to respond to abscission signals. Ethylene promotes and auxin inhibits this step by reducing the effect of ethylene. During this step, the AZ forms two layers: a proximal separation layer (pink circles) and a distal layer (white circles). In Arabidopsis flowers, AZ maturation requires TALE homeodomain transcription factors. In step 3, separation occurs as hydrolytic enzymes released from cells in the separation layer degrade the cell wall and middle lamella, causing the organ to shed. Ethylene initiates this process, with the IDA–HAE/HSL2 signalling pathway required for completion. In step 4, exposed AZ cells differentiate into epidermal cells that secrete a protective layer. The IDA signalling pathway, important for this process, also triggers a defence response in AZs to protect against infection during sealing. (B) AZ anatomy. The AZ contains secession cells (SECs) that detach and residuum cells (RECs) that remain on the plant. SECs include proximal and distal AZ cells. RECs transdifferentiate into a protective epidermal layer. (C) Comparison of Arabidopsis floral organ AZs (top) and tomato pedicel AZs (bottom). Both have proximal separation layers, but their distal layers differ. Arabidopsis has a lignified honeycomb structure that focuses enzymes secreted by the separation layer. *IDA* is expressed in SECs, whereas its receptors, HAE/HSL2, are present in both SECs and RECs. This suggests that diffusion of the IDA peptide induces formation of the protective layer. In tomato, the distal layer undergoes programmed cell death. The domains of *IDA* and *HAE/HSL2* expression are unknown. For simplicity, only the key regulators of AZ development are shown at each step.

Abscission involves four overlapping steps (Patterson, 2001; Taylor and Whitelaw, 2001; Roberts *et al.*, 2002) and (Fig. 3). In step 1, the AZ is initiated when a band of meristematic cells forms at a selected location in the plant, generally an organ boundary. In step 2, AZ cells become competent to respond to abscission signals. This step is triggered when auxin levels decrease in AZ cells, making them sensitive to ethylene. In parallel, the AZ differentiates further into a proximal domain of cells specialized for secretion and a distal domain of cells at the base of the leaving organ, each contributing to the separation process. In step 3, cell separation is executed when hydrolytic enzymes are released into the break plane between the organ and plant body. This step is promoted by ethylene and requires a signalling pathway led by INFLORESCENCE DEFICIENT IN ABSCISSION (IDA), a secreted peptide that binds to a receptor complex on the surface of AZ cells. In step 4, a protective layer is formed, which includes the synthesis of a new epidermis onto the surface of the AZ (Addicott, 1982; Patterson, 2001; Sundaresan *et al.*, 2020).

### Step 1: initiation

**Fig. 4.**
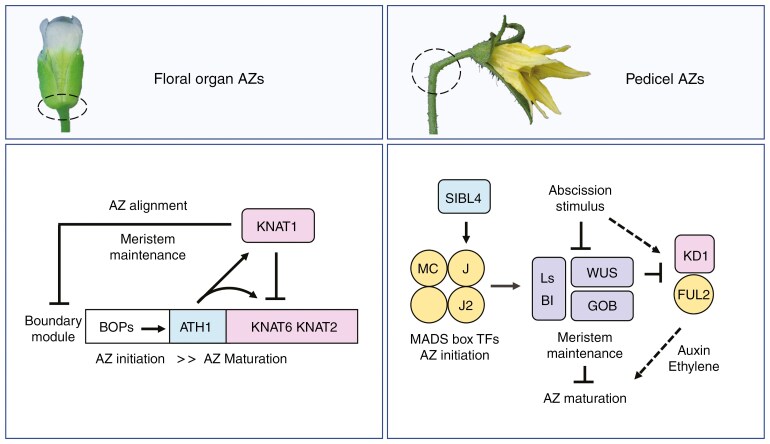
Abscission zone (AZ) formation in Arabidopsis and tomato. (Left) Arabidopsis floral organ AZs. Boundary genes contribute at multiple steps. BOP1 and BOP2 are essential for AZ initiation. TALE homeodomain factors ATH1, KNAT6 and KNAT2 promote AZ initiation and are required for AZ maturation. KNAT1 represses this module to position the AZ and prevents premature abscission. ATH1 plays a key regulatory role as it activates the expression of other members of this module. (Right) Tomato pedicel AZs. MADS-box proteins are essential for AZ initiation and upregulate factors that promote meristem activity. Abscission signals downregulate these factors, leading to AZ maturation and cell separation. Tomato BEL4 (SlBL4) promotes *J* expression. Under low light, WUS negatively regulates *KD1* and *FUL2*, which modulate hormone pathways to promote pedicel abscission.

In Arabidopsis, the formation of cauline and floral organ AZs requires BLADE-ON-PETIOLE (BOP) 1 and 2 co-activators (McKim *et al.*, 2008; Figs 3A and 4). The *bop1 bop2* double mutant lacks AZ cells and consequently fails to abscise (McKim *et al.*, 2008). BOP1 and BOP2 belong to a larger NPR1 (NON-EXPRESSOR OF PATHOGENESIS-RELATED GENES 1) protein family involved in plant defence. This family is divided into three clades containing BTB/POZ (Bric-a-Brac/POX virus and Zinc finger) domain and ankyrin repeats (Backer *et al.*, 2019). BOP1 and BOP2 regulate boundary development (Khan *et al*., 2014) but also contribute to defence by forming complexes with TGACG-motif binding (TGA) bZIP transcription factors (Hepworth *et al.*, 2005; Wang *et al.*, 2019; Zhang *et al.*, 2019). In boundaries, BOP1 and BOP2 require the downstream activity of three amino-acid-loop-extension (TALE) homeodomain transcription factors (Khan *et al.*, 2012, 2015; Hepworth and Pautot, 2015). The TALE family contains KNOTTED-like (KNOX) and BELL1-like (BELL) members that function in heterodimers (Hamant and Pautot, 2010; Hay and Tsiantis, 2010). TALE members have functions in shoot apical meristem and boundary maintenance, but they also contribute to abscission at different steps. The MYB transcription factor ASYMMETRIC LEAVES 1 (AS1) represses the transcription of *KNOTTED-LIKE FROM ARABIDOPSIS 1* (*KNAT1*), also named *BREVIPEDICELLUS* (*BP*), to align AZs properly at the boundary (Gubert *et al.*, 2014), whereas KNAT1 mutation leads to AZ enlargement and premature abscission (Wang *et al.*, 2006; Shi *et al.*, 2011*b*). This defect is associated with a higher, prolonged expression of *KNAT6* and *KNAT2* in AZ cells, two TALE members that function antagonistically to KNAT1 to prevent premature abscission (Shi *et al.*, 2011*b*). Double mutation of *KNAT6* and *KNAT2* slightly disrupts AZ structure and organ shedding (Shi *et al.*, 2011*b*; Crick *et al.*, 2022). Loss of ARABIDOPSIS HOMEOBOX GENE 1 (ATH1), a KNAT6 partner, results in disorganized AZs and delayed stamen shedding (Gómez-Mena and Sablowski, 2008). When *knat6* and *knat2 knat6* mutations are combined with *ath1* mutation, AZ initiation is delayed and floral organ abscission abolished (Crick *et al.*, 2022). The triple mutant flowers have early boundary defects, including organ fusions, and lack small characteristic AZ cells. In a *knat1 bop1 bop2* mutant background where *ATH1*, *KNAT6* and *KNAT2* expression is elevated, AZ initiation is partly recovered, indicating a contribution of these TALE factors in AZ initiation (Crick *et al.*, 2022).

The F-box protein HAWAIIAN SKIRT (HWS) also regulates AZ development. In *hws* mutants, floral organs are fused at their bases and abscission is delayed. An AZ forms, but pectinase *POLYGALACTURONASE ABSCISSION ZONE A. THALIANA* (*PGAZAT*) gene expression is reduced and delayed (González-Carranza *et al.*, 2017). These phenotypes were rescued in *hws* mutants by expressing microRNA 164-resistant versions of the transcription factors CUP-SHAPED COTYLEDON 1 or 2, which confer boundary identity (González-Carranza *et al.*, 2017), suggesting that boundary identity might be necessary for full responsiveness to abscission signals.

In tomato, petals and stamens are shed from AZs shortly after pollination (Lanahan *et al.*, 1994; Okabe *et al.*, 2011; Xu *et al.*, 2016). CRISPR-Cas9 knockout of three *BOP* homologues blocks this abscission (Xu *et al.*, 2016; Izhaki *et al.*, 2018). These homologues interact with ALOG (Arabidopsis LIGHT-DEPENDENT SHORT HYPOCOTYLS 1 and Oryza G1) transcription factors. Mutations in the ALOG family member TERMINATING FLOWER (TMF) prevent leaf and floral organ abscission, implicating a role for BOP-ALOG complexes in AZ formation (Izhaki *et al.*, 2018). CRISPR mutants for Arabidopsis ALOG homologues are now available for further study (Rieu *et al.*, 2024).

In tomato pedicels, quaternary complexes containing the MADS-box transcription factors JOINTLESS (J), MACROCALYX (MC) and SEPALLATA-like JOINTLESS-2 (also known as SlMBP21) are essential for AZ formation (Mao *et al.*, 2000; Zhang *et al.*, 2000; Liu *et al.*, 2014; Gomez Roldan *et al.*, 2017; Figs 3A and 4). Mutations in any of these factors lead to a *jointless* phenotype (Zhang *et al.*, 2000). The *jointless-2* (*j-2*) trait, which keeps the sepals and pedicel attached to the plant, is valuable in agriculture because the ‘stem-less’ fruit facilitate mechanical harvesting (Butler, 1936; Zahara and Scheuerman, 1988). At an early stage of AZ initiation, all three MADS-box genes are co-expressed in vascular tissue in the pedicel core (Liu *et al.*, 2014). Their activities promote the expression of meristem fate regulators WUSCHEL (LeWUS), GOBLET (GOB), LATERAL SUPPRESSOR (Ls) and BLIND (Bl), indicating that AZ cells are in an indeterminate state before abscission (Nakano *et al.*, 2012, 2013; Gomez Roldan *et al.*, 2017). As abscission nears, AZ cells enlarge (Sexton and Redshaw, 1981; Tabuchi *et al.*, 2001) and decrease in expression of *Ls*, *GOB* and *LeWUS*, indicating a change in their state (Nakano *et al.*, 2013).

In tomato, BEL1-LIKE HOMEODOMAIN 4 (SlBL4), an orthologue of Arabidopsis ATH1, contributes to AZ development by directly activating *JOINTLESS* (Yan *et al.*, 2021). *SlBL4* RNAi mutants have enlarged pedicel AZs with extra epidermal cell layers. These mutants also show changes in the expression of shoot meristem, auxin-related and cell wall hydrolytic enzyme genes, consistent with defects in AZ patterning and early abscission (Yan *et al.*, 2021), highlighting related roles for ATH1-like TALE members in AZ development (Gómez-Mena and Sablowski, 2008; Crick *et al.*, 2022).

Altogether, these studies show that AZs can be formed by different mechanisms depending on their location. BOPs play an essential role at floral organ boundaries in Arabidopsis and tomato flowers, whereas MADS-box factors govern AZs in tomato pedicels. MADS-box factors are also required for fruit DZ and seed AZ formation in Arabidopsis plants (Liljegren *et al.*, 2000; Pinyopich *et al.*, 2003; Balanzà *et al.*, 2016). BELL and KNOX regulators function in both systems, emphasizing the meristematic nature of AZs. Future research should investigate the downstream targets of these two patterning systems to discover whether there are common denominators.

### Step 2: competence

**Fig. 5.**
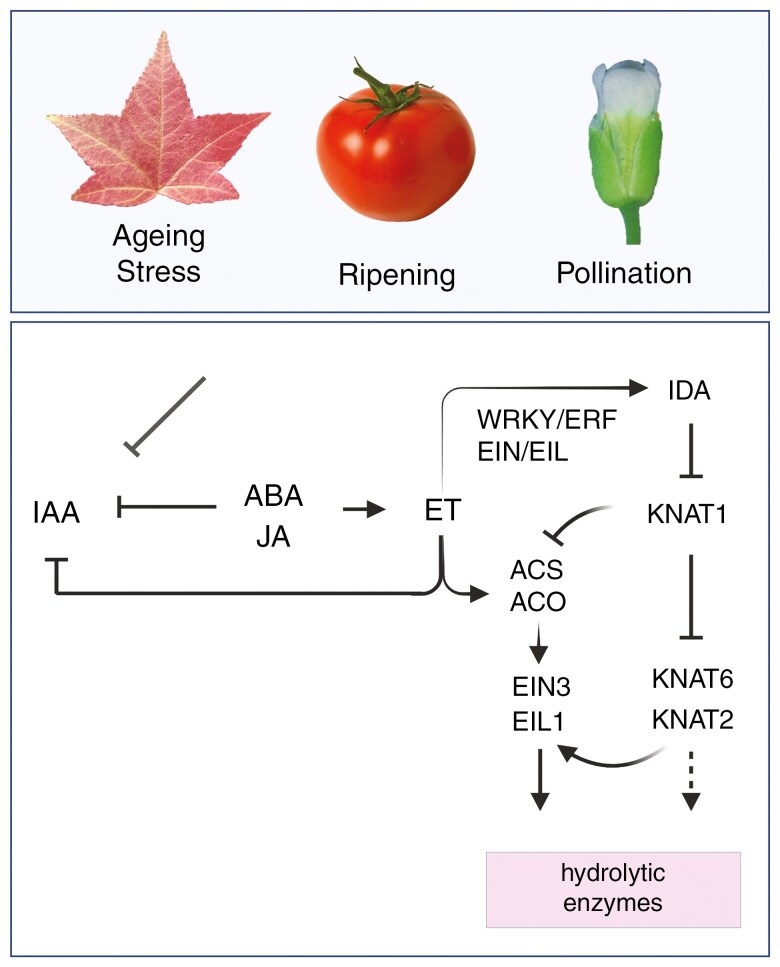
Model for ethylene (ET) and IDA signalling integration. ABA, JA and ET produced in response to stress or natural processes accelerate abscission by inhibiting IAA biosynthesis, transport and catabolism and promoting ET production. In litchi, ET upregulates EIN/EIL orthologues that activate genes involved in ET biosynthesis and cell wall hydrolysis. *IDA* transcription is regulated by EIN/EIL factors (litchi) and ERF/WRKY factors (*Arabidopsis*). KNAT1 represses the ET pathway (litchi) and negatively regulates KNAT6/KNAT2 function (*Arabidopsis*). IDA signalling inhibits KNAT1 activity, which enhances ET biosynthesis and promotes cell separation.

In step two, AZ cells become responsive to abscission signals (Figs 3A and 5). Arabidopsis floral organs are shed a few days after pollination (Patterson, 2001), whereas tomato fruit pedicel abscission is induced by ripening (Roberts *et al.*, 1984; Ito and Nakano, 2015). Senescence and environmental stresses, including temperature extremes, drought or water-logging, high or low light, resource limitation, and wounding or infection can also induce abscission (Gulfishan *et al.*, 2019; Patharkar and Walker, 2019; Ma *et al.*, 2021), showing that AZs integrate diverse stimuli to regulate separation.

Abscisic acid (ABA) was characterized initially as the main inducer of fruit and leaf shedding but later found to promote abscission by increasing ethylene levels (Cracker and Abeles, 1969). The role of ethylene in abscission was established at the turn of the century, following its discovery as the active component of illuminating gas, whose leakage caused street trees to shed their leaves (Jackson and Osborne, 1970; Meir *et al.*, 2019). The ratio between auxin and ethylene plays a key role. Auxin acts as a negative regulator, and its decline initiates the abscission process, whereas ethylene acts as an accelerator and promotes cell separation (Meir *et al.*, 2015).

#### Depletion of auxin, the trigger signal.

Auxin depletion in AZ cells is the main trigger for abscission (Meir *et al.*, 2015). A steady flow of auxin from distal organs to the AZ prevents abscission, but its reduction sensitizes the AZ to ethylene (Meir *et al.*, 2015). Growing organs with high auxin levels do not abscise (Addicott, 1982). With ageing or stress, auxin production declines, while hormones that respond to these signals, mainly ethylene, but also ABA and jasmonic acid (JA), increase (Addicott, 1991). Ethylene promotes abscission by inhibiting auxin transport to the AZ and by reducing free auxin in AZ cells (Taylor and Whitelaw, 2001; Basu *et al.*, 2013; Meir *et al.*, 2015).

Auxin is perceived in the cytosol by TIR/AFB subunits of an SCF (SKP1–Cullin–F-box) ubiquitin ligase complex. Auxin binding promotes TIR1/ABF interaction with AUX/IAA repressor proteins, leading to their ubiquitination and proteolysis. Degradation of AUX/IAA proteins releases auxin response factors (ARFs) to regulate auxin-responsive gene expression (Leyser, 2018).

In Arabidopsis, the auxin influx carriers AUXIN RESISTANT1 (AUX1), LIKE AUX1 (LAX1) and LAX3 are expressed in AZ cells. Mutants in these carriers shed their floral organs one or two positions earlier than normal (Basu *et al.*, 2013). Auxin levels in AZ cells directly impact abscission timing, as shown by transgenic plants expressing bacterial auxin (IAA) biosynthetic (*iaaM*) or auxin catabolic (*iaaL*) genes in the AZ leading to petal loss that was delayed or accelerated, respectively (Basu *et al.*, 2013). Mutations in ARF1 and ARF2, both expressed in AZs, also delay abscission, with ARF2 loss having a greater effect (Ellis *et al.*, 2005; Okushima *et al.*, 2005). The *arf1* mutation delays abscission by increasing the expression of Aux/IAA genes in flowers (Ellis *et al.*, 2005), whereas the *arf2* mutation reduces ACC synthase gene expression (Okushima *et al.*, 2005). These findings suggest that coordinated regulation between auxin and ethylene triggers the abscission response in AZ cells.

Auxin dynamics in AZ cells remain unexamined by fluorescent reporters or biosensors, leaving questions about how signals such as pollination influence auxin transport, ethylene accumulation and the timing of AZ development. In the Arabidopsis gynoecium, fertilization increases auxin at the valve margin, promoting cell division. PIN-FORMED 3 (PIN3) auxin efflux carriers are reduced in the plasma membrane, concentrating auxin to form the DZ (van Gelderen *et al.*, 2016). Later in fruit development, an auxin minimum at the DZ is needed to form the separation layer (Sorefan *et al.*, 2009). Thus, an early accumulation of auxin drives cell division for cell layer formation, and its later depletion promotes lignification and cell separation for pod shattering (Ballester and Ferrándiz, 2017). These auxin dynamics are controlled by the valve margin identity bHLH transcription factor INDEHISCENT (Sorefan *et al.*, 2009; van Gelderen *et al.*, 2016). Transcription factors involved in AZ development might play a similar role.

In tomato, SlPIN1 regulates auxin efflux and the ethylene sensitivity of AZ cells (Shi *et al.*, 2017). Silencing *SlPIN1* decreases auxin transport from the ovary to AZ cells, leading to accelerated pedicel abscission. Tomato *ARF* genes, homologous to Arabidopsis abscission-related *ARF*s (*AtARF1*/*SlARF1* and *AtARF2*/*SlARF2*/*SlARF11*) are expressed dynamically in pedicel AZs, but their functions are uncharacterized (Guan *et al.*, 2014). However, downregulating tomato *miRNA160*, which targets *SlARF10*/*SlARF16*/*SlARF17*, impairs petal and ripe fruit abscission (Damodharan *et al.*, 2016).

Studies analysing transcriptional changes in tomato pedicel AZs after flower removal identified two phases of response to auxin depletion. Early responses (0–4 h after flower removal) included the downregulation of genes in  auxin transporter (PIN and AUX/LAX), auxin response (SAUR and GH3) and auxin transcription factor (Aux/IAA and ARF) families, confirming changes in auxin metabolism (Meir *et al.*, 2015). Late responses (8–14 h after flower removal) included the upregulation of genes from ethylene biosynthesis (ACS and ACO) and transcription factor (ERF) families and AZ-related genes encoding cell wall-modifying enzymes (Meir *et al.*, 2010). Early events occurred normally when plants were treated with an ethylene blocker (1-Methylcyclopropene), showing distinct responses to auxin depletion and ethylene increase, whereas auxin application after flower removal blocked both phases, showing that auxin functions as a master regulator (Meir *et al.*, 2010, 2015).

Tomato KNOTTED1-LIKE HOMEODOMAIN PROTEIN1 (KD1) controls abscission by regulating auxin levels and response in pedicel AZs (Ma *et al.*, 2015). A KNATM member lacking a homeodomain, KD1 is likely to interact with other TALE proteins to control their activity. Overexpression of KD1 accelerates abscission, whereas silencing delays it (Ma *et al.*, 2015). Silenced lines show alterations in auxin-related genes, but also in other pathways, indicating that KD1 activity is complex (Sundaresan *et al.*, 2021). SlBEL11, expressed in the fruit AZ, prevents premature fruit drop by regulating auxin transport into pedicels. It induces SlMYB111, which upregulates flavonoid production, including quercetin, which inhibits auxin transport to modulate the auxin gradient and abscission timing (Dong *et al.*, 2024).

#### Ethylene, the execution signal.

Ethylene is produced in response to fertilization, in ripening fruit and senescing floral organs (Meir *et al.*, 2019). The hormone is sensed by receptors in the endoplasmic reticulum membrane, including ETHYLENE RESPONSE 1 (ETR1). Upon ethylene detection, the membrane protein ETHYLENE-INSENSITIVE 2 (EIN2) is cleaved, releasing a cytosolic fragment that moves into the nucleus. This action indirectly stabilizes the transcription factors EIN3 and ETHYLENE-INSENSITIVE 3-LIKE 1 (EIL1), which are key regulators of ethylene responses (Binder, 2020).

In Arabidopsis, the ethylene-insensitive mutants e*tr1-1* and *ein2-1* show a marked delay in floral organ shedding and the activation of hydrolytic enzymes, indicating that ethylene is important but non-essential for cell separation (Bleecker and Patterson, 1997; Patterson and Bleecker, 2004; Ogawa *et al.*, 2009). Likewise, *ein3 eil1* double mutants show delayed abscission (Ma *et al.*, 2020). On the contrary, the ethylene-overproducing mutant, *eto4*, sheds its organs slightly earlier than normal (Sundaresan *et al.*, 2015). Flowers treated with ethylene shed their organs even before fertilization, showing that AZs are receptive even before flowers reach maturity (Butenko *et al.*, 2003).

Tomato plants engineered to produce high levels of ethylene undergo premature senescence and flower abscission, often before anthesis (Lanahan *et al.*, 1994). The tomato ethylene receptor (ETR) family has six members, including LeETR3/NEVER RIPE (NR) (Zhou *et al.*, 1996; Wilkinson *et al.*, 1997; Lashbrook *et al.*, 1998; Tieman and Klee, 1999; Tieman *et al.*, 2001). Plants impaired for ethylene perception or sensitivity show delayed fruit ripening and pedicel abscission (Lanahan *et al.*, 1994; Tieman *et al.*, 2001; Whitelaw *et al.*, 2002; Okabe *et al.*, 2011). The LeETR receptors interact with a CC-type tetratricopeptide repeat protein (SlTPR1) acting as a Hsp70/Hsp90 co-chaperone, which, when overexpressed, inhibits AZ formation and abscission in both tomato and Arabidopsis plants (Lin *et al.*, 2008; Prasad *et al.*, 2010).

#### Environmental stresses.

Environmental stresses such as shading, drought, water-logging, temperature extremes and pathogen infection can induce organ abscission (Taylor and Whitelaw, 2001; Patharkar and Walker, 2019). Abiotic stresses increase ABA levels, leading to the synthesis of 1-aminocyclopropane-1-carboxylic acid (ACC), a precursor molecule, which is transported to distal tissues and converted into ethylene by ACC synthase (Cracker and Abeles, 1969; Gómez-Cadenas *et al.*, 1996). JA also contributes to abscission, in part by stimulating ethylene production (Taylor and Whitelaw, 2001; Wang *et al.*, 2021*b*).

JA pathway mutants show delayed abscission but shed earlier in response to ethylene than non-treated plants (Ogawa *et al.*, 2009; Kim *et al.*, 2013*a*, *b*; Jibran *et al.*, 2017). The *ein2* mutant abscises earlier with methyl JA treatment, and the *ein2 allene oxide synthase* (*aos*) double mutant shows a greater delay in abscission than either single mutant (Kim *et al.*, 2013*b*). The *aba deficient2* (*aba2*) mutant abscises normally, but further delays the abscission of an *ein2 aos* double mutant, showing an impact of all three hormones on abscission (Ogawa *et al.*, 2009; Lee *et al.*, 2022). Methyl JA can also induce fruit drop in tomato and fleshy fruits (Rohwer and Erwin, 2008).

#### Arabidopsis cauline leaf abscission.

The shedding of Arabidopsis cauline leaves under stress involves mechanisms similar to floral organ abscission (Patharkar and Walker, 2016). Drought-wilted plants that are re-watered shed their cauline leaves after 1–2 days. The ethylene-insensitive mutants, *ein2-1* and *ein2-5*, do not shed in these conditions, highlighting the crucial role of ethylene (Meir *et al.*, 2022). ABA pathway mutants (*abi1*, *aba1* and *aba2*) still undergo abscission, showing a lesser role (Patharkar and Walker, 2016). Cauline leaves treated with *Pseudomonas syringae* also abscise, a response requiring intracellular detection of the pathogen, because a bacterial mutant lacking the delivery system for effectors did not show this response (Patharkar *et al.*, 2017).

#### Flower drop in tomato.

Stresses such as drought, temperature, nutritional deficiency or low light cause flower drop in fleshy fruits (Patharkar and Walker, 2019; Ma *et al.*, 2021). In tomato, low light increases *Sl*CLAVATA3 peptide, which binds to transmembrane receptors in the pedicel AZ, repressing *SlWUS*. This repression upregulates *SlKD1* and *SlFRUITFULL2*, altering the auxin gradient and increasing ethylene production (Cheng *et al.*, 2022). A similar feedback loop in the shoot apical meristem (Brand *et al.*, 2000; Schoof *et al.*, 2000) emphasizes the suppression of meristem activity during abscission.

Low light reduces photosynthesis, decreasing soluble sugars and starch in leaves and flowers. Starch breakdown in flowers supplies temporary carbon that inhibits flower loss. This remobilization is potentially regulated by sucrose non-fermenting-like kinase (SnRK1) in response to low trehalose 6-phosphate (T6P) and high ABA levels (Li *et al.*, 2022*a*). In litchi fruits starved for carbohydrate, upregulated LcHB2/LcHB3 HD-ZIP proteins activate the ABA biosynthetic gene *LcNCED3* and ethylene biosynthesis genes *LcACS1*/*4*/*7* and *LcACO2*/*3* to promote abscission (Li *et al.*, 2015, 2019*a*).

Phytosulfokine (PSK) peptides control flower drop under drought stress, in addition to their function in plant immunity (Reichardt *et al.*, 2020). Drought induces *PSK1* and *PSK6* expression in the AZ, where subtilisin-like phytaspase2 cleaves PSK precursors into mature mPSK peptides. These peptides bind to PSK receptors 1 and 2, repressing genes that keep the AZ inactive and activating the expression of cell wall hydrolases, promoting flower drop (Reichardt *et al.*, 2020). Phytaspase2 activity is negatively regulated by a protease inhibitor (*Sl*PI26), whose activity is repressed in drought conditions (Wang *et al.*, 2025).

#### Reactive oxygen species.

Reactive oxygen species (ROS) are a convergence for abscission pathways, released by stressed or damaged cells (Petrov *et al.*, 2015) or produced during normal development (Ma *et al.*, 2021). ROS release reduces auxin by inhibiting its transport and increasing its conjugation, which induces ethylene (Meir *et al.*, 2015). ROS treatments accelerate leaf and fruit abscission in a variety of species, whereas antioxidants or ROS scavengers inhibit abscission (Meir *et al.*, 2015). In cotton, thidiazuron application led to H_2_O_2_ accumulation and leaf abscission that was partly suppressed by the inhibition of NADPH oxidase (RBOH) enzymes (Li *et al.*, 2022*b*). In litchi, DOF (DNA binding one finger) transcription factor LcDOF5.60 works together with LcRBOHD to control fruitlet abscission (Ma *et al.*, 2023).

In Arabidopsis, CYCLING DOF FACTOR 4 (CDF4) promotes ABA production and suppresses H_2_O_2_ scavenging, resulting in ROS accumulation during floral organ abscission (Xu *et al.*, 2020*a*). The MSD2 manganese superoxide dismutase also regulates ROS levels. Loss-of-function mutants show earlier abscission, characterized by superoxide accumulation and the activation of *IDA* signalling genes. These genes, more rapidly upregulated in response to ABA and nitric oxide in *mds2* mutants, emphasize the role of ROS in regulating the timing of abscission (Lee *et al.*, 2022).

### Step 3: separation

As abscission approaches, the AZ forms distinct distal and proximal cell layers (Fig. 3). Ethylene and the IDA–HAE/HSL2 signalling pathway drive the separation process (Fig. 5). Separation starts with cell expansion and loosening of the cell wall (a complex network of cellulose microfibrils, hemicellulose and pectin), followed by degradation of the middle lamellae. Enzymes activated during this step include cell wall loosening proteins and degrading enzymes (Addicott, 1982; Sexton and Roberts, 1982).

#### AZ maturation.

Single-cell RNA sequencing in Arabidopsis identified two cell populations in the AZ: residuum cells (RECs) located in the receptacle (that are retained); and secession cells (SECs) located at the edge of the abscising organ (that secede) (Lee *et al.*, 2018; Fig. 3B). RECs accumulate superoxide (O_2_^−^), whereas SECs accumulate hydrogen peroxide (H_2_O_2_) (Lee *et al.*, 2018). These ROS profiles are involved in regulating cell fate, similar to the ROS gradients in meristems that balance stem cell maintenance and differentiation (Qin, 2023). SECs differentiate into two cell layers: distal cells, at the edge of the separating organ, form a lignified layer, whereas proximal cells, next to the receptacle, form a separation layer (Fig. 3C). Distal lignified cells form a rigid brace that focuses the hydrolytic enzymes released by proximal cells in the separation layer (Lee *et al.*, 2018). The honeycomb shape of brace cells results from the localization of laccases and peroxidases at cell corners during lignification (Hoffmann *et al.*, 2020). RECs sequester cell wall processing enzymes internally to escape digestion (Lee *et al*., 2018). When the organ detaches, exposed RECs on the surface of the receptacle form a new epidermis with a protective cuticle (Lee *et al.*, 2018).

Lignification of distal cells is preceded by ROS accumulation, produced by RESPIRATORY BURST (RBOH) oxidases in the plasma membrane and cell wall peroxidases or laccases. ROS production requires BOP1/BOP2 and involves RBOHD and RBOHF (Lee *et al.*, 2018; Crick *et al*., 2022). In their absence, ROS levels are reduced, and a lignin brace fails to form, although separation occurs normally (Crick *et al.*, 2022; Lalun *et al.*, 2024). A lignified layer in the fruit DZ is more crucial, as shown by a mutation in *NAC SECONDARY WALL THICKENING PROMOTING FACTOR 1*, which causes indehiscent siliques (Mitsuda and Ohme-Takagi, 2008). A lignified layer is also essential for rice shattering but not in many grasses (Yu and Kellogg, 2018, 2024). In *Eragrostis tef*, for instance, shattering relies on cell fracture and programmed cell death (PCD) (Yu *et al.*, 2023).

TALE factors also control the precise architecture of AZ cell layers. KNAT1/BP negatively regulates the size and number of AZ cells (Shi *et al.*, 2011*b*). An *enhancer of bp 1* (*ebp1*) mutant was isolated, showing delayed abscission and abnormal SECs and RECs. SECs display a concave surface, and RECs are abnormally stacked (Yun *et al.*, 2024). Transcriptomic analysis showed that *ebp1* SECs have REC-like characteristics and an increase in expression of SEPARATION AFFECTING RNA-BINDING PROTEIN1 (SARP1), a protein involved in formation of the separation layer, which might inhibit BOP2 by RNA binding together with or downstream of KNAT1/BP (Yun *et al.*, 2024).

Likewise, ATH1 with KNAT6 and KNAT2 control AZ structure. In the *ath1 knat2 knat6* triple mutant, the AZ is expanded and disorganized. Boundaries between the different cell layers are impaired. Lignin is not deposited, and the expression of genes associated with cell separation is reduced (Crick *et al.*, 2022). These findings delineate two phases of AZ development: *bop1 bop2* mutations block AZ initiation, whereas *ath1 knat6 knat2* mutations highlight a second phase of AZ development that forms lignified and separation layers immediately before abscission.

In tomato pedicel AZs, distal cells undergo PCD instead of forming a lignin brace (Fig. 3C). Cell death begins in the pedicel core, accompanied by an enrichment of NADPH oxidase and ROS in distal AZ cells (Bar-Dror *et al.*, 2011). Two PCD nuclease genes, *LX* and *TBN1*, are expressed asymmetrically in distal AZ cells (Bar-Dror *et al.*, 2011; Chersicola *et al.*, 2017). Inhibition of LX or overexpression of an anti-apoptotic protein delayed abscission, supporting the role of PCD in pedicel abscission (Lers *et al.*, 2006; Bar-Dror *et al.*, 2011). Treatment with 1-Methylcyclopropene blocked cell separation, but affected PCD to only a moderate extent, indicating that ethylene is not essential for distal AZ processes (Chersicola *et al.*, 2017).

Autophagy plays a role in Arabidopsis petal abscission (Furuta *et al.*, 2024). JA produced in the anthers at dehiscence signals to petal bases, inducing a chromatin state that activates autophagy. This switch involves JA-responsive MYC transcription factors that enhance chromatin accessibility for transcription factors such as ANAC102, which induces ROS and autophagy-related genes. Mutants with reduced autophagic activity, such as *atg5* and *atg7*, showed delayed petal abscission (Furuta *et al.*, 2024). So far, this process is not widely implicated.

#### Cell expansion.

Immediately before separation, the internal pH of cells in the separation zone becomes alkaline, and the apoplast acidifies. Protons released to the cell wall activate pH-sensitive cell wall modifying proteins, such as pectinases and expansins, causing cell expansion (Sexton and Roberts, 1982; Osborne and Morgan, 1989; Sundaresan *et al.*, 2015; Cosgrove, 2024).

In tomato, cell expansion is mediated by tonoplast intrinsic protein SlTIP, an aquaporin residing in the plasma membrane and tonoplast of pedicel AZ cells (Wang *et al.*, 2021*a*). SlTIP promotes abscission by raising cytoplasmic H_2_O_2_ levels and water permeability. Elevated H_2_O_2_ reduces auxin signalling and increases ethylene production, creating a feedback loop with the transcription factor SlERF52. SlERF52 enhances aquaporin expression (Wang *et al.*, 2021*a*) and upregulates genes involved in cell wall hydrolysis, facilitating cell separation (Nakano *et al.*, 2014).

#### Dissolution of the middle lamella.

The primary cell wall consists of cellulose microfibrils embedded in a matrix of hemicelluloses, pectins and structural proteins. Xyloglucan, a major hemicellulose, non-covalently cross-links cellulose strands for strength (Cosgrove, 2024). The pectin-rich middle lamella binds adjacent cells together, with homogalacturonan as the main pectin and with smaller amounts of branched rhamnogalacturonan I and II (Cosgrove, 2024). Grasses can differ from dicots in the types and abundance of hemicelluloses, pectins, xyloglucans and aromatic compounds (Calderan-Rodrigues *et al.*, 2019; Penning *et al.*, 2019). In some grasses, such as *Brachypodium distachyon*, AZ cells are more lignified than neighbouring cells (Yu *et al.*, 2020*b*). How these differences affect cell wall processing remains unknown.

Various cell wall degrading enzymes and remodelling proteins are involved in abscission (Roberts *et al.*, 2000; Agustí *et al*., 2008; Cai and Lashbrook, 2008; Meir *et al.*, 2010; Corbacho *et al.*, 2013; Li *et al.*, 2015; Tsuchiya *et al.*, 2015; Glazinska *et al.*, 2017; Merelo *et al.*, 2017; Lee *et al.*, 2018; Cosgrove, 2024). Endo-1,4-β-glucanases (cellulases) and xyloglucan endotransglucosylase/hydrolases (XTH) digest cellulose and hemicellulose in the primary cell wall. Expansins, activated at low pH, loosen cell wall adhesion by disrupting non-covalent bonding between cellulose microfibrils and xyloglucans. β-Galactosidases remove terminal β-d-galactosyl residues from hemicellulose and branched pectins, and esterases cleave methyl or acetyl groups from galacturonic acid residues to change cell wall mechanical properties. Pectin lyases and polygalacturonases hydrolyse the glycosidic bonds between galacturonic acid residues in pectin chains.

The importance of these enzyme classes has been demonstrated in various studies (e.g. Brummell *et al.*, 1999; Cho and Cosgrove, 2000; Tsuchiya *et al.*, 2015; Merelo *et al.*, 2017). Polygalacturonases are especially important, because they degrade the middle lamella. Silencing tomato polygalacturonases (TAPGs) delayed pedicel abscission (Jiang *et al.*, 2008), and silencing *PGAZAT/ADPG2* in Arabidopsis delayed floral organ loss (González-Carranza *et al.*, 2007, 2017). At least three polygalacturonases (ADPG1, ADPG2 and QRT2) are required for normal floral organ abscission and fruit dehiscence (Ogawa *et al.*, 2009).

Arabinogalactans influence cell wall mechanics (Cosgrove, 2024). In Arabidopsis floral AZs, soluble arabinogalactan proteins are secreted during and after abscission (Stenvik *et al.*, 2006; Cho *et al.*, 2008; Crick *et al.*, 2022). When abscission is incomplete, loosened organs will reattach owing to the continuous rebuilding of cell walls (Addicott, 1982; Butenko *et al.*, 2003; Cho *et al.*, 2008; Liu *et al.*, 2013). Another cell wall glycoprotein, tomato hybrid proline-rich protein (THyPRP), has a signalling role. Its silencing delays abscission by reducing AZ cell sensitivity to ethylene (Sundaresan *et al.*, 2018).

#### Secretion.

Vesicle trafficking delivers enzymes and cell wall material to the AZ during abscission. Transmission electron microscopy studies in various species show morphological changes, such as the expansion of the endoplasmic reticulum and Golgi apparatus, in response to ethylene or natural abscission (Addicott, 1982; Bar-Dror *et al.*, 2011; Chersicola *et al.*, 2017). In tomato AZs, vesicles multiply and fuse to form larger vesicular structures, including paramural bodies, located between the plasma membrane and the cell wall. Highly branched plasmodesmata connect adjacent cells during this phase. As abscission progresses, cells on the proximal side of the AZ separate by gradual dissolution of the middle lamella. Cells remaining on the plant retain a highly developed endomembrane system for protective layer formation (Bar-Dror *et al.*, 2011; Chersicola *et al.*, 2017).

In Arabidopsis, abscission is abolished by mutations in the ADP-ribosylation and GTPase activating protein NEVERSHED (NEV), which is essential for cargo trafficking in the trans-Golgi network and endosomes (Liljegren *et al.*, 2009). Transmission electron microscopy analysis of *nev* sepal AZs revealed abnormal Golgi structures, displacement of the trans-Golgi network, and excess of paramural vesicles between the plasma membrane and the cell wall (Liljegren *et al*., 2009). Additional membrane-associated receptor-like kinases, CAST AWAY and EVERSHED (SOBIR1), play less understood roles in regulating vesicle transport during abscission (Leslie *et al.*, 2010; Burr *et al.*, 2011; Taylor *et al.*, 2019).

NEV homologues in tomato are uncharacterized, but a recent study identified three main categories of vesicle trafficking-related proteins (GTPases, SNAREs and SNARE regulators) expressed in AZs after tomato flower removal (Sundaresan *et al.*, 2020). Functional analysis of these proteins might help to clarify how specific types of cargo are trafficked in vesicles for cell separation, rebuilding the cell wall as part of a new epidermis, then depositing a protective layer.

#### The IDA signalling pathway.

**Fig. 6.**
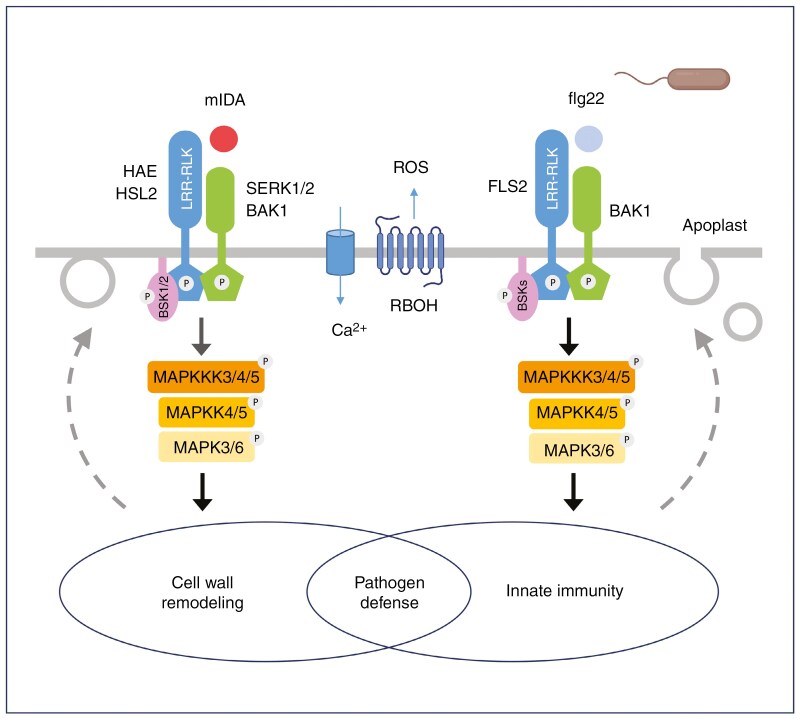
Overlaps between IDA and innate immunity signalling pathways. (Left) When mIDA binds, a complex between HAE/HSL2 and its co-receptor BAK1 (and other SERKs) is formed. BSKs are phosphorylated to initiate signal transduction, including ROS production, a Ca^2+^ burst and the induction of an MAPK cascade. These signals lead to abscission and the induction of pathogen defence genes associated with innate immunity. (Right) When flg22 binds, a complex between FLS2 and its co-receptor, BAK1, is formed. BSKs are phosphorylated, initiating the same signalling events and resulting in an innate immunity response.

In Arabidopsis, the IDA signalling pathway is essential for cell separation (Shi *et al.*, 2019; Figs 3–6). The core components of this pathway were identified through mutants that retained their floral organs (Butenko *et al.*, 2003; Cho *et al.*, 2008).

The pathway begins with the secreted peptide IDA, which binds to transmembrane leucine-rich repeat receptor-like kinases called HAESA (HAE) and HAESA-LIKE2 (HSL2) (Butenko *et al.*, 2003; Stenvik *et al.*, 2006, 2008; Cho *et al.*, 2008). The IDA propeptide is processed by subtilisin-like proteases into its active mIDA form, a 14-amino-acid peptide (Schardon *et al.*, 2016). Hydroxylation of a central proline in mIDA peptide increases its activity 30-fold (Butenko *et al.*, 2014). The activated mIDA binds to a canyon-shaped pocket on HAE/HSL2 receptors (Santiago *et al.*, 2016), causing them to pair with SOMATIC EMBRYOGENESIS RECEPTOR KINASE 1-2-3-4 (SERK) family kinases, which trans-phosphorylate each other (Meng *et al.*, 2016; Santiago *et al.*, 2016).

The signal is relayed by a mitogen activated protein kinase (MAPK) cascade involving MAPKKK3/4/5 (Wang *et al.*, 2022), MAPK kinases MKK4/5, and MAPKs MPK3/6 to initiate cell separation (Cho *et al.*, 2008). This cascade is tightly regulated to prevent cell damage, a potential risk for infection (Taylor *et al.*, 2024).

MAP KINASE PHOSPHATASE 1 (MKP1) negatively regulates MPK3 and MPK6 (Ulm *et al.*, 2002; Bartels *et al.*, 2009) and functions as a negative regulator of HAE/HSL2 signalling (Taylor *et al.*, 2024). Mutations in *MPK1* partly restore abscission in *hae hsl2* mutants, indicating that MPK1 might suppress basal levels of MAPK activity that could otherwise trigger abscission without HAE/HSL2 activation (Taylor *et al.*, 2024).

AGAMOUS LIKE 15 (AGL15) and AGL18 also inhibit abscission (Fernandez *et al.*, 2000; Patharkar and Walker, 2015). AGL15 targets the *HAE* promoter. During IDA signalling, MPK3 and MPK6 phosphorylate AGL15 at serine 231 and 257 (Patharkar *et al.*, 2016) inhibiting its activity, leading to a rapid increase in *HAE* expression immediately before cell separation (Patharkar and Walker, 2015). AGL15 and AGL6 also form complexes with FOREVER YOUNG FLOWER/AGL42 and FOREVER YOUNG FLOWER-LIKE 1/AGL71, delaying abscission in part by downregulating *IDA/HAE* expression in AZs (Chen *et al.*, 2011, 2022).

AtDOF4.7 is another inhibiting factor of abscission, a target of MPK3/6 phosphorylation *in vitro* (Wang *et al.*, 2016). AtDOF4.7 interacts with ZINC FINGER PROTEIN 2, a negative regulator of abscission (Cai and Lashbrook, 2008; Wei *et al.*, 2010) and directly represses the *PGAZAT/ADPG2* gene, suggesting that its activity targets hydrolytic enzymes (Wei *et al.*, 2010). However, many *in vivo* MAPK substrates remain unidentified.

Brassinosteroid signalling kinases (BSKs), including BSK1, BSK2 and, to a lesser extent, BSK3, activate the MAPK cascade (Galindo-Trigo *et al.*, 2024*b*). BSK1 forms a complex with HAE/HLS2 receptors, controlling abscission independently of its kinase activity. This complex is believed to recruit MAPKKK4/YODA (YDA), which interacts with BSK1 in other developmental processes and immunity (Galindo-Trigo *et al.*, 2024*b*). MAPKKK4/YDA is a positive regulator of abscission downstream of *IDA* and *HAE/HSL2*, whereas BSK1 activity depends on *HAE/HSL2* to rescue abscission in *ida* mutants. Disrupting YDA in AZ cells in a *mapkkk3 mapkkk5* double mutant or a wild-type background causes similar abscission defects, placing YDA as the lead MAPKKK during floral organ abscission.

Plants overexpressing IDA have larger AZs and shed floral organs earlier than normal. Abscission of cauline leaves, siliques and flowers and early dehiscence of silique valves can occur (Stenvik *et al.*, 2006). In *ida* mutants, abscission is incomplete and organs remain attached (Butenko *et al.*, 2003). Recessive *knat1* alleles were discovered to restore abscission in *ida* and *hae hsl2* mutants. Similar to *35S:IDA* plants, *knat1* mutants show enlarged AZs and earlier floral organ shedding (Wang *et al.*, 2006; Shi *et al.*, 2011*b*).

KNAT1 controls inflorescence growth by restricting *KNAT6* and *KNAT2* expression to boundary regions (Ragni *et al.*, 2008; Zhao *et al.*, 2015) and, likewise, prevents premature abscission by repressing these genes in AZs. IDA signalling inhibits KNAT1, thereby increasing *KNAT6* and *KNAT2* expression in AZs to promote cell separation (Shi *et al.*, 2011*b*). KNAT1 also restricts *BOP1/BOP2*/*ATH1* expression to AZs, but unlike *KNAT6* and *KNAT2*, their expression is unaffected in *ida* mutants (Crick *et al.*, 2022). Overexpression of *BOP2* or *BOP1*, restores abscission in *ida* mutants, probably by upregulating *KNAT6* and *KNAT2* (Crick *et al.*, 2022). BOP1–TGA complexes directly activate *ATH1* and indirectly activate *KNAT6* and *KNAT2* (Khan *et al.*, 2015; Wang *et al.*, 2019). Reduced polygalacturonase and cellulase transcript levels in *bop1 bop2* and *ath1 knat6 knat2* AZs support this mechanism (Crick *et al.*, 2022). Thus, the expression of *KNAT6* and *KNAT2* in AZs is regulated by both BOP1/BOP2/ATH1 and the IDA signalling pathway.

KNAT1 and KNAT2 or KNAT6 might regulate abscission by opposing effects on ethylene (Hamant *et al.*, 2002; Zhao *et al.*, 2020; Fig. 5). In litchi, *LcKNAT1* expression decreases in the fruitlet AZ during abscission. LcKNAT1 directly represses ethylene biosynthesis genes (Zhao *et al.*, 2020) and *LcEIL2* and *LcLEIL3* to prevent premature abscission (Tieman *et al.*, 2001; Ma *et al.*, 2020).

#### IDA/IDL peptides.

The *ida* mutant has a weak abscission defect, with floral organs loosely retained during early fruit elongation (Butenko *et al.*, 2003). This reflects roles for other IDA-like (IDL) peptides in AZs (Butenko *et al.*, 2003; Vie *et al.*, 2015). Of the seven *IDL* genes in Arabidopsis, *IDL*, *IDL2*, *IDL3* and *IDL4* are expressed in floral receptacles (Stenvik *et al.*, 2008; Vie *et al.*, 2015). Overexpression of *IDA1*, *IDL2*, *IDL3*, *IDL4* and *IDL5* induces early abscission, but only *IDL1*, when expressed under the *IDA* promoter, restores abscission in an *ida* mutant, suggesting a more central role, linked to its homology with the C-terminal EPIP-C domain (extended-PIP-C) of IDA (Stenvik *et al.*, 2008; Galindo-Trigo *et al.*, 2024*a*). A quadruple mutant (*ida idl1 idl2 idl3*) retains its floral organs more strongly than the *ida* mutant, confirming genetic redundancy among these peptides (Galindo-Trigo *et al.*, 2024*a*).

Tomato plants have 11 *SlIDA* and at least eight *SlHAE* genes (Li *et al.*, 2021; Lu *et al.*, 2023). Expression analysis of pedicel AZs identified five *SlIDA* genes (*SlIDA*, *SlIDL2*, *SlIDL3*, *SlIDL4* and *SlIDL5*) and two *SlHSL* genes *(SlHSL6* and *SlHSL7*) associated with natural abscission (Lu *et al.*, 2023). Mature *SlIDA* peptides have been detected *in vivo* (Wang *et al.*, 2020; Li *et al.*, 2021). Ten *PROLYL 4-HYDROXYLASE* (*P4H*) genes are also found in the tomato genome. Silencing of *SlP4H3* delayed pedicel abscission in ripe fruits by reducing cell wall hydrolases, implying that proline hydroxylation of SlIDA peptides is crucial for their function (Perrakis *et al.*, 2019).

*IDA* or *IDA*-like genes are present in bryophytes and all studied angiosperms, including soybean, citrus, litchi, yellow lupine, tobacco, mango, rose and oil palm (Tucker and Yang, 2012; Estornell *et al.*, 2015; Stø *et al.*, 2015; Ying *et al.*, 2016; Wilmowicz *et al.*, 2018; Olsson *et al.*, 2019; Shi *et al.*, 2019; Guo *et al.*, 2021; Rai *et al.*, 2021; Ventimilla *et al.*, 2021; Singh *et al.*, 2023; Wang *et al.*, 2023). Heterologous expression of *IDA* genes from citrus (*CtIDA3*), litchi (*LcIDL1*), mango (*MiIDA1* and *MilDA2*) and rose (*RbIDL1* and *RbIDL4*) in Arabidopsis restores abscission in *ida* mutants (Estornell *et al.*, 2015; Ying *et al.*, 2016; Rai *et al.*, 2021; Singh *et al.*, 2023), suggesting broad functional conservation of the IDA–HAE/HSL2 pathways across species.

#### IDA and ethylene.

The IDA signalling pathway is upregulated by ethylene but has distinct functions (Jinn *et al.*, 2000; Butenko *et al.*, 2003; Meir *et al.*, 2019; Fig. 5). For example, *ida* mutants treated with ethylene have a normal triple response but fail to abscise (Butenko *et al.*, 2003). Conversely, *IDA* overexpression activates dormant AZs in cauline leaves and pedicels, a response that ethylene alone cannot induce (Stenvik *et al.*, 2006).

IDA activation by ethylene is supported by a narrower domain of *IDA* expression in the *etr1-1* mutant (Butenko *et al.*, 2006). The *IDA* promoter has four ethylene response elements (EREs), and mutating one of these EREs drastically reduced its activity in floral organ AZs, suggesting that EREs are crucial for *IDA* expression (Galindo-Trigo *et al.*, 2024*a*).

Ethylene induces the AZ-specific expression of *IDA* homologues in various species, including tomato, soybean, citrus, oil palm, yellow lupine, mango and litchi (Tucker and Yang, 2012; Estornell *et al.*, 2015; Wilmowicz *et al.*, 2018; Tranbarger *et al.*, 2019). Conversely, its antagonist, 2,5-norbornadiene, delays *IDL* gene expression in soybean (Tucker and Yang, 2012).

In lupine, the application of IDA peptide to floral AZs induced the formation of numerous plasmodesmata and vesicle structures, suggesting an increased metabolic activity (Bar-Dror *et al.*, 2011; Wilmowicz *et al.*, 2022). These changes, similar to those induced by ethylene treatment, suggest that basal amounts of ethylene might induce *LlIDL* gene expression, producing bioactive peptides that further stimulate ethylene production, ultimately promoting floral AZ activation and organ detachment (Wilmowicz *et al.*, 2022).

In litchi, ethylene is shown to complement IDA signalling pathways. Produced in response to ethylene, LcEIL2 and LcEIL3 promote fruitlet abscission by directly activating genes involved in ethylene biosynthesis (*LcACS/LcACO*) and cell wall degradation (*LcPG1/LcPG2* and *LcCEL2/LcCEL8*) (Ma *et al.*, 2020). The ethylene-induced HD-ZIP transcription factor LcHB2 also directly activates *LcCEL2* and *LcCEL8* by binding to promoter elements (Li *et al.*, 2019*b*). Another ethylene-induced transcription factor, LcERF2, represses pedicel growth and accelerates fruit drop by inhibiting genes involved in cell wall carbohydrate metabolism and inducing genes involved in cell wall degradation (Yi *et al.*, 2021), whereas LcERF10 accelerates abscission by increasing the cytosolic pH of fruitlet AZs cells through the direct repression of *LcNHX7*, which encodes an Na^+^–H^+^ exchanger (He *et al.*, 2023). Other ethylene-induced transcription factors upregulate the IDA signalling pathway (Ma *et al.*, 2024). LcARF5 directly activates *LcIDL1* and *LcHSL2*, and LcEIL3 directly activates *LcIDL1* (Ma *et al.*, 2024). Silencing *LcARF5* and *LcEIL3* reduces *LcIDL1* and *LcHSL2* expression in fruitlet AZs, suggesting that LcARF5–LcIDL1/LcHSL2 and LcEIL3–LcIDAL1 modules play a role in sensing the auxin–ethylene balance in litchi fruitlet AZs and convert these cues into molecular events that promote cell separation.

In Arabidopsis, *IDA* and *HAE* transcripts peak at stage 15 before separation (Cai and Lashbrook, 2008; Niederhuth *et al.*, 2013; Patharkar and Walker, 2015). RNA sequencing of wild-type and *hae hsl2* stage 15 flower receptacles revealed HAE/HSL2 signalling dependence for genes involved in pectin degradation, cell wall remodelling and extracellular barrier formation, encompassing the processes of both RECs and SECs (Niederhuth *et al.*, 2013; Lee e*t al.*, 2018).

Cell type-specific expression studies and single-cell RNA sequencing highlight the distinct metabolic roles of RECs and SECs (Lee *et al.*, 2018; Taylor *et al.*, 2024). *IDA* expression is specific to SECs, whereas *HAE*/*HSL2* are expressed in both domains (Taylor *et al.*, 2024; Fig. 3C). SECs are enriched for transcripts related to lignin biosynthesis and cell wall modification, whereas RECs are enriched for transcripts associated with epidermal cell fate and cuticle formation (Lee *et al.*, 2018; Taylor *et al.*, 2024). The specific localization of IDA to SECs of abscising organs is proposed to terminate HAE/HSL2 signalling after organ separation (Taylor *et al.*, 2024).

Transcriptomic analyses of AZs show that many hydrolase and cell wall remodelling genes do not depend on the IDA–HAE/HSL2 pathway, suggesting that additional pathways, such as ethylene, contribute to cell separation (Niederhuth *et al.*, 2013; Kim *et al.*, 2015). The relative contributions of ethylene and IDA signalling pathways to the regulation of cell separation genes is still not well documented and might be species specific (Tucker and Yang, 2012; Lee *et al.*, 2018; Meir *et al.*, 2019; Taylor *et al.*, 2024).

#### IDA and stress signals.

WRKY transcription factors co-ordinate stress-induced abscission. In Arabidopsis, WRKY57 acts as a positive regulator of *IDA* and *IDA*-like genes in response to treatment with flagellin22 (flg22) peptide. The *IDA* promoter contains five WRKY binding sites, suggesting that this activation is direct (Galindo-Trigo *et al.*, 2024*a*). Floral organ abscission in response to WRKY57 requires both IDA and HAE/HSL2 receptors, linking plant immunity to abscission (Galindo-Trigo *et al.*, 2024*a*).

In tomato, low light and ethylene promote abscission by activating SlWRKY17, a direct positive regulator of *SlIDL6* (Li *et al.*, 2021). SlIDL6 signalling increases cytosolic Ca^2+^ levels, stabilizing the calcium-dependent protein kinase SlCPK10, which further promotes abscission (Fu *et al.*, 2024). Knockdown of PSK signalling enhances flower drop in *SlIDL6* knockout lines, and co-application of PSK and IDL6 peptides synergistically accelerates abscission (Li *et al.*, 2021). Further research is needed to understand the interaction of these two peptide signalling pathways.

### Step 4: sealing

In the final step of abscission, a new epidermis is formed to protect the AZ surface from water loss and infection (Patterson, 2001; Lee *et al.*, 2018). Pathogens such as olive knot and stone fruit canker are known to enter leaf scars (Hewitt, 1938; Crosse, 1951; Cao *et al.*, 2013). Slow sealing cultivars of peach and cherry are also prone to infection (Addicott, 1982).

#### Protective layers and diversity.

In Arabidopsis, RECs become epidermal cells that secrete a protective cuticle (Lee *et al.*, 2018). Epidermal cell identity is typically established during embryogenesis and maintained throughout the life of the plant (Javelle *et al.*, 2011; Lee *et al.*, 2018). Understanding this shift in cell fate during abscission is a key future direction.

Cuticle-associated genes are significantly affected in *hae hls2* mutants, indicating that their activation is a key role of the IDA–HAE/HSL2 pathway (Niederhuth *et al.*, 2013; Kim *et al.*, 2015; Taylor *et al.*, 2024). A comparative transcript analysis in Arabidopsis, tomato and soybean revealed a surprisingly early increase in cutin- and wax-associated genes in AZs, suggesting that the cuticle might provide a smooth surface in aiding cell separation or organ detachment (Kim *et al.*, 2015). Supporting this, plants silenced for three AP2-like transcription factors, SHINE1/2/3, have fused floral organs and organ dispersal defects linked to a reduced cutin load and modified cell wall properties (Shi *et al.*, 2011*a*). Fused floral organs are also common in cuticle mutants of Arabidopsis and tomato (Ingram and Nawrath, 2017; Petit *et al.*, 2021).

Suberin, or cork, forms in the periderm, the outer bark that replaces the cuticle in woody plants (Vishwanath *et al.*, 2015). Protective layers with suberin or lignin are found in tomato pedicel AZs (Tabuchi *et al.*, 2000, 2001) and in woody species such as chestnut, poinsettia, cotton, peach, citrus and cherry, where a periderm forms (Addicott, 1982; Biggs and Northover, 1985; Agustí *et al.*, 2008; Cao *et al.*, 2013). The variable composition of the protective layer across species indicates that its formation is a differentiation process, as opposed to a wounding response.

#### Protective layers and immunity.

Pathogenesis-related genes are activated during cell separation, suggested to protect AZs from infection during sealing (Coupe *et al.*, 1997; Cai and Lashbrook, 2008; Meir *et al.*, 2011; Niederhuth *et al.*, 2013; Kim *et al.*, 2015; Lee *et al.*, 2018). In *hae hsl2* mutants, defence genes are downregulated, showing the importance of the IDA–HAE/HSL2 signalling pathway in this response (Kim *et al.*, 2015; Taylor *et al.*, 2024; Fig. 6).

In addition to their role in abscission, *IDA* and *IDL* genes are induced by stress stimuli, including flg22 and chitin, which elicit pattern-triggered immunity (Vie *et al.*, 2015, 2017; Galindo-Trigo *et al.*, 2024*a*; Lalun *et al.*, 2024). In Arabidopsis seedlings, mIDA peptide treatment causes a HAE/HSL2 receptor-dependent cytosolic Ca^2+^ burst and extracellular ROS release, in common with immune responses (Lalun *et al.*, 2024). Likewise, in tobacco leaves, mIDA triggers ROS production in the presence of HAE and HSL2 receptors (Butenko *et al.*, 2014).

Both IDA and pattern-triggered immune signalling involve similar components, including LRR-RLK receptors, BAK1/SERK co-receptors, BSKs and MAPK cascades, which activate genes that modify the cell exterior (Patharkar and Walker, 2019; Wang *et al.*, 2022; Galindo-Trigo *et al.*, 2024*b*; Lalun *et al.*, 2024). mIDA treatment in seedlings induces defence-related marker genes, such as *FRK1*, *MYB51* and *PEP3*, in common with flg22 (Lalun *et al.*, 2024). Co-treatment of seedlings with IDA and flg22 further enhances these responses, showing their distinct but complementary effects. Unlike flg22, which elicits lignin and callose deposition, IDA triggers a response specific to AZs. Emphasizing the critical nature of this pathway, the accumulation of pathogenesis-related mRNAs during leaflet abscission in peppers does not require ethylene (Coupe *et al.*, 1997).

In parallel, BOP–TGA complexes in AZs might contribute to the activation of defence genes, as shown in leaves and stems of cotton (Zhang *et al.*, 2019). The interplay between abscission and plant defence, and how these responses are integrated, remains under-explored. These defence genes might have been co-opted from early land plants to control cell separation, a process that includes immune signalling to protect exposed cells.

## MANIPULATING ABSCISSION

Studies of model systems, such as Arabidopsis and tomato, have clarified the regulatory mechanisms of abscission. The next challenge is to apply this knowledge to reduce crop losses. Emerging technologies, including CRISPR gene editing, show great promise, but only a few varieties developed through these approaches have reached commercialization (Prado *et al.*, 2024). The final section of this review highlights recent advancements in this field.

### Abscission zone differentiation

Inhibition of factors involved in AZ differentiation shows promise for crops such as vegetables or cut flowers, where abscission during ripening and senescence is detrimental. Many commercial varieties of tomato contain a *jointless* mutation that facilitates mechanical harvesting. However, this mutation in large-fruited varieties causes undesirable inflorescence branching and a higher rate of fruit imperfections (Gomez Roldan *et al.*, 2017; Soyk *et al.*, 2017; Tonutti *et al.*, 2023; Huerga-Fernández *et al.*, 2024). This issue was resolved in two field tomato varieties by using CRISPR-Cas9 to edit *j-2* alleles. The edited lines have both a jointless pedicel and normal inflorescence architecture (Soyk *et al.*, 2019). Field trials confirmed that two of the edited lines, created in a relatively short time frame, performed as well as conventionally bred lines (Lee and Hutton, 2021). Thus, targeting genetic components of fruit or flower AZ differentiation can be used to manage abscission separately from ripening and senescence.

KNOX-BELL genes are also targets for breeding. In rice, the KNAT1 homologue OSH15 interacts with BELL-like proteins, qSH1 and OsSH5. OSH15–qSH1 pairs are required for AZ initiation, whereas OSH–SH5 pairs inhibit lignin deposition in the AZ to regulate seed shattering. Mutations in these genes lead to non-shattering rice plants (Yoon *et al.*, 2017; Zhang *et al.*, 2022). CRISPR-Cas9 editing of qSH1 was used to produce non-shattering homozygous lines, which were then bred to create hybrid rice varieties with desirable intermediate seed shattering traits, demonstrating a viable strategy for adjusting abscission traits (Sheng *et al.*, 2020; Wu *et al.*, 2022)

### Abscission zone competence

Targeting ethylene production has been effective in extending shelf life and inhibiting abscission in tomato and melon (Shipman *et al.*, 2021). In melon, CRISPR/Cas-9 mutations in an NAC domain transcription factor that promotes ethylene biosynthesis genes delayed ripening and abscission without changing fruit quality (Liu *et al.*, 2022). Highly perishable flowers are often transported long distances to market, even before reaching the consumer (Shipman *et al.*, 2021). In carnation, targeting ethylene biosynthesis to lower its production nearly doubled vase life (Savin *et al.*, 1995). In petunia, editing to mutate the *ACO1* gene increased flower longevity by 6–10 days (Xu *et al.*, 2020*b*). With the genomes of >69 ornamental plant species now completed, the targeted regulation of abscission traits through gene editing has become an attainable goal (Zheng *et al.*, 2021).

### Abscission zone activation

Drugs in human medicine are used to alter biological processes. A similar approach in plants involves the use of peptides or small molecules to modulate the IDA signalling pathway. The C-terminal region of IDA-like peptides contains a conserved 12-amino acid motif (PIP) that is crucial for activity. Synthetic peptides containing this motif induce flower abscission in yellow lupine (Wilmowicz *et al.*, 2021). An extended version of this minimal peptide (EPIP) can stimulate organ separation in oil palm and poplar (Tranbarger *et al.*, 2019).

In *Brassica napus*, CRISPR-Cas9 editing of two *IDA*-like genes produced a double mutant that retained its petals. This retention significantly reduced fungal infection by *Sclerotinia sclerotiorum* and silique shattering during harvest, two major contributors to yield loss (Geng *et al.*, 2022; Wu *et al.*, 2022). The extended flowering period of the double mutant is also desirable in regions where the crop is a tourist attraction owing to its bright yellow flowers (Geng *et al.*, 2022; Wu *et al.*, 2022).

Enzymes are also promising targets for genetic engineering. In tomato, silencing three AZ-specific polygalacturonases (TAPG1, TAPG2 and TAPG4) reduced leaf and fruit drop (Kalaitzis *et al.*, 1997; Jiang *et al.*, 2008; Sundaresan *et al.*, 2019). Subtilisin proteases involved in PSK peptide processing also show potential. Tomato plants silenced for Phyt2 retained about twice as many flowers under drought, leading to higher fruit set (Reichardt *et al.*, 2020; Chandrasekaran *et al.*, 2025).

### Abscission zone protective layers

Enhancing the protective layer in crops might improve disease resistance. Leaf scars are entry points for bacterial or fungal infections in fruit trees, such as peach, apple, pear, kiwi and cherry, which are susceptible to cankers. In peach and pear trees, thicker protective layers are correlated with canker resistance (Feliciano and Daines, 1970; Biggs and Northover, 1985). The higher expression of the pathogenesis-related 5 gene during fruit ripening in European plum increased resistance to the necrotrophic fungus *Monilinia fructicola* (El-kereamy *et al.*, 2011). Thus, targeting genes that strengthen the protective layer through the IDA signalling pathway might prove useful.

## THE FINAL CUT

Model systems, such as Arabidopsis and tomato, have significantly expanded our knowledge of the cellular dynamics of AZs. Abscission is understood to have core mechanisms that are conserved yet flexible. The location, structure and activation of AZs are finely tuned to benefit the plant. There is no universal set of transcription factors for building an AZ. Once formed, a depletion of auxin followed by sensitivity to ethylene are conserved steps that sequentially form a competent AZ. A layered structure is common, but its components are variable. There is always a separation layer, but there are different degrees of digestion that can be complete or partial, sometimes requiring programmed cell death or mechanical forces to finish the process. The final stage of separation involves a conserved peptide signalling pathway with homology to defence reactions of the innate immune system. This pathway produces hydrolytic enzymes and pathogenesis-associated proteins that complete cell separation and synthesize a new epidermis at the break plane, which protects against infection and water loss. The protective coating that forms depends on the species, ranging from a simple cuticle to a woody periderm.

Future abscission research presents both challenges and opportunities. One key area is to translate findings from dicots to cereals, where seed shattering traits are crucial for yield. In grasses, the role of MADS-box or BOP orthologues in AZ initiation is unclear (Yu *et al.*, 2020*a*, *b*, 2023; Li and Su, 2024; Yu and Kelloggs, 2024). Various other transcription factors, including BELL, KNOX, YABBY, AP2 and WRKY members, control abscission, often by influencing lignin deposition (Yu and Kellogg, 2018, 2024). In barley, BRITTLE RACHIS 1 and 2, potentially a receptor–ligand pair, determine grain shattering (Pourkheirandish and Komatsuda, 2022; Yu and Kellogg, 2024). Meanwhile, the IDA–HAE/HSL2 signalling pathway is relatively unexplored. For instance, the rice genome contains three *IDA*-like genes whose functions are unknown (Wang *et al.*, 2023). Many grass genes involved in seed shattering map more closely to the DZ in Arabidopsis fruits (Dong and Wang, 2015; Yu *et al.*, 2020a; Wang *et al.*, 2023). Exploring these areas will shed light on how abscission pathways have diverged across species.

Although core mechanisms for abscission have been identified, the mechanics of stress-induced abscission, a major source of crop loss, requires further study. This includes clarifying the role of peptide hormones, whose signalling contributes to both abscission and immune responses. IDA/IDL and PSK peptides share many of the same components for signal transduction, raising the possibility of both co-operative and inhibitory effects on abscission and immune pathways (Zhang *et al.*, 2022; Wang *et al.*, 2023).

Critical questions also remain about how AZ sites are selected and how their development is coordinated with processes such as dehiscence, injury or predation. Advances in single-cell transcriptomics, hormone biosensors and live cell imaging now offer tools to examine these processes at single-cell resolution.

What started as mere curiosity about the defoliating effects of illuminating gas on trees is steadily leading to transformative advancements in crop science, thanks to benchmark studies in model plants.
